# Autoinducer-2 Quorum Sensing Influences Viability of *Escherichia coli* O157:H7 under Osmotic and *In Vitro* Gastrointestinal Stress Conditions

**DOI:** 10.3389/fmicb.2017.01077

**Published:** 2017-06-13

**Authors:** Hyunjoon Park, Kyuyeon Lee, Soyoung Yeo, Heuynkil Shin, Wilhelm H. Holzapfel

**Affiliations:** ^1^Department of Advanced Green Energy and Environment, Handong Global UniversityPohang, South Korea; ^2^Research Institute of Eco-friendly Livestock Science, Institute of Green-Bio Science and Technology, Seoul National UniversityPyeongchang, South Korea; ^3^School of Life Science, Handong Global UniversityPohang, South Korea

**Keywords:** EHEC, quorum sensing, autoinducer-2, gastrointestinal stress, osmotic stress, bacterial survival

## Abstract

Bacteria use autoinducer molecules to communicate both at intra-species and inter-species levels by quorum sensing. One such cell density-dependent signaling system is the *luxS*-mediated universal quorum sensing using autoinducer-2 (AI-2). Virulence of several pathogens is determined by an AI-2 system and is related to colonization and infection of the host. From this concept, numerous papers have suggested that AI-2 inhibition is an important strategy toward designing of new antimicrobial agents. However, recent studies indicate that the AI-2 system is also involved in adaptation and survival under environmental stress conditions. Therefore, we hypothesized that interaction between quorum sensing and environmental conditions may be critical in influencing predicted results in a control and when combating of target pathogens. We investigated the growth of enterohemorrhagic *Escherichia coli* O157:H7 (EHEC) and its *luxS*-deficient (non AI-2 producing) mutant strain under various stress conditions, and found significant differences in the growth rate under osmotic stress. Moreover, we could also show the impact of the AI-2 molecule on viability in the gastrointestinal tract model representing a complex environmental condition. Differences in vital responses of the strains suggest that AI-2 quorum sensing has a significant influence on the viability of EHEC under environmental stress conditions.

## Introduction

Quorum sensing, a secretory bacterial communication system, regulates cell-density dependent behavior with regard to the expression of a specific set of genes determining social behavior (Miller and Bassler, 2001; Winzer and Williams, 2001; Hammer and Bassler, 2003; Leung and Lévesque, 2012; García-Contreras et al., 2014). Autoinducer-1 (AI-1) quorum sensing is referred to as an intra-species signaling feature (March and Bentley, 2004), with the autoinducer-2 (AI-2) system being proposed to be an inter-species signaling system (Federle and Bassler, 2003). The *luxS* gene encoded AI-2 synthase inter-converts AI-2 molecules from 4,5-dihydroxy-2,3-pentanedione (DPD), a 5-carbon precursor (Xavier et al., 2007). Several pathogenic bacteria also use quorum sensing to regulate virulence factors; thus, interference with quorum sensing is being considered as a new strategy for alternative antibiotics with target specificity (Finch et al., 1998; Rasmussen and Givskov, 2006; Rasko and Sperandio, 2010). In theory, it is assumed that the cell density dependent feature of quorum signal systems, when, associated with signaling absence, do not interfere with bacterial growth or viability; it is therefore expected that quorum signaling inhibition or quenching strategies could avoid resistance of a pathogen (Otto, 2004).

Based on the potential advantages for combating pathogens, quorum sensing inhibitors (QSIs) have been intensively studied both for medical applications and food safety (Hentzer et al., 2003; Smith and Iglewski, 2003; Medellin-Peña et al., 2007; Park et al., 2014). However, recent reports opened questions on potential advantages and implied a need to investigate hitherto unveiled characteristics of QSIs. First, the expected anti-pathogenicity of QSIs can be incapacitated. Bacteria can develop resistance to QSIs by multiple quorum sensing systems, mutation, efflux systems, or environmental conditions (Defoirdt et al., 2010; Kalia et al., 2014). Secondly, unlike the previous theory, quorum sensing interference can affect bacterial viability. Recent fundamental research has unveiled novel roles of the quorum sensing systems to impact not only collective signaling but also global regulation of bacterial physiology (Lee et al., 2013; van Kessel et al., 2015; Thompson et al., 2015). Especially, studies on the involvement of AI-2 quorum sensing in stress-response have provided evidences for a significant impact on bacterial growth, survival, metabolism, adaptation, and colonization (Lebeer et al., 2007, 2008; Moslehi-Jenabian et al., 2009; Christiaen et al., 2014; Sun et al., 2015).

Enterohemorrhagic *Escherichia coli* serotype O157:H7 (EHEC) is a foodborne pathogen of worldwide public health concern (Nguyen and Sperandio, 2012). EHEC colonizes the human colon epithelium where it induces acute colonic inflammation at A/E lesions constructed by type III secretion system (T3SS) leading to hemolytic-uremic syndrome (HUS) by endotoxin (Shiga-toxin) production (Garmendia et al., 2005; Pacheco and Sperandio, 2012). EHEC also has a LuxS/AI-2 signaling system for expression of its virulence factors (Sperandio et al., 1999). However, there is only sparse information on its impact on EHEC growth or vital mechanisms under gastrointestinal conditions. In this research, we investigated the AI-2 mediated differences in expression of stress response and virulence factors of EHEC under various stress conditions, and studied the impact of AI-2 on EHEC survival using *in vitro* and *in vivo* gastrointestinal tract models.

## Materials and Methods

### Bacterial Strains and Culture Conditions

Enterohemorrhagic *E. coli* O157:H7 was obtained from the ATCC under the strain number 43894; its *luxS*-deficient strain was described in our previous study (Park et al., 2014). *E. coli* strains were stored at -80°C in Luria-Bertani (LB) broth (BD Difco, United States) with 20% glycerol added and grown at 37°C in the LB broth. The strains were sub-cultured three times at 37°C before use. The strains and all related expendables were autoclaved at 120°C for 20 min before disposal. For stress response observation, pH, NaCl, bile, temperature, glucose (limitation), anaerobic conditions were, respectively, used as single stress factor. pH was adjusted by using HCl (5M) and NaOH (10N). Anaerobic experiments were performed in an anaerobic chamber (Coy Laboratory Products, Ann Arbor, MI, United States) with an atmosphere consisting of 5% CO_2_, 10% H_2_, and 85% N_2_. Bacterial growth was measured at OD 600 nm using a SPECTROstar nano spectrophotometer (BMG Labtech, Germany). For ATP detection, a BacTiter-Glo Microbial Cell Viability Assay kit (Promega, United States) was used following the manufacturer’s instructions. The ATP was measured by a GloMax^®^ 96 Microplate Luminometer (Promega, United States). (S)-4,5-dihydroxy-2,3-pentanedione (OMM Scientific, United States) was used as synthesized AI-2 molecule.

### Field Emission Scanning Electron Microscopy (FE-SEM)

Bacterial cells were collected by centrifugation at 10,000 rpm, and washed three times with PBS. The cell pellets were fixed in a 2.5% glutaraldehyde solution (Sigma–Aldrich, United States) for 2 h. Then, pellets were washed three times with PBS, and post-fixation performed in a 1% osmium tetroxide solution for 1 h. Then the pellets were dehydrated with a series of increasing ethanol concentrations, and the slides coated with platinum. The cells were observed with a Field Emission Scanning Electron Microscope (FE-SEM) 8700F Prime (JEOL Ltd., Japan) in the National Center for Inter-University Research Facilities (Seoul National University, South Korea). To calculate individual cell length-to-width parameters, IC measure (The Imaging Source Co., Ltd) free-software was used.

### Transcriptional Analysis (Microarray)

Total RNA of tested strains was extracted at late-log phase in normal LB broth and 0.6M NaCl LB broth using RNeasy Mini kit (Qiagen, Germany). Under the osmotic stress conditions the OD_600__nm_ values of the wild-type and mutant strains for RNA extraction were 0.15–0.25 and 0.55–0.65, respectively. GeneChip E. coli Genome 2.0 Array (Affymetrix, United States) microarray platform was used. cDNA was synthesized using the GeneChip 3’IVT Plus Reagent Kit as described by the manufacturer. After Biotin-labeling, amplified RNA was synthesized from 100 ng total RNA using the 3’IVT Plus Reagent Kit. A 12 μg labeled cRNA was fragmented by heat and ion-mediated hydrolysis at 94°C for 35 min. The fragmented cRNA was hybridized for 16 h at 45°C in a hybridization oven. Hybridized arrays were obtained using a GeneChip Fluidics Station 450 and a GCS3000 Scanner (Affymetrix, United States). Array data export processing and analysis were performed using Affymetrix^®^ GeneChip Command Console^®^ Software R 3.0.2.

### Gastrointestinal Tract (GIT) Assay

The GIT *in vitro* imitation assay was based on a modification of the model by Weiss and Jespersen (2010), with composition and concentration of each liquid substrate (“juice”) following the model. 10^9^ CFU/ml of EHEC wild-type and *luxS* mutant strains were, respectively, centrifuged and suspended in 2 mL of saliva juice. After 5 min incubation, 2.4 mL of the gastric juice were added and incubated for 1 h. Then, 2.4 mL of duodenum juice, 1.2 mL of bile juice (concentrations according to the model), and 0.4 mL of NaHCO_3_ (1M) were added and followed by incubation for 2 h at 37°C. After GIT assay, the strains were harvested for ATP detection.

### Mouse *In Vivo* Experiments

This study was carried out in accordance with the guidelines set forth by the Korean Association for Laboratory Animals. The protocol was approved by the Committee on the Ethics of Animal Experiments of Handong Global University.

Infection EHEC wild-type and *luxS* mutant strains was induced by oral administration (10^9^ CFU per mouse) to the 4 weeks old male ICR mice (Daehan Bio Link Co., Ltd., South Korea) receiving laboratory chow diet feeding *ad libitum*. After 8 h, each mouse intestinal tract was extracted and homogenized in 1:9 volume of PBS. The samples were centrifuged at 1,500 rpm for 10 min, and the supernatant filtered by 100, 70, 40, and 10 μm pore size of syringe filter, gradually. In order to detect the EHEC strains in the sample, FITC conjugated *E. coli* O157 monoclonal antibodies (Thermo Scientific, United States) were diluted 20-fold and used to combine with each sample (1:1). After 30 min incubation at 37°C, samples were washed two times with PBS at 12,000 rpm for 3 min. Samples were detected by Infinite 200 Pro multimode reader (Tecan, Switzerland).

### Statistical Analysis

The experimental data were analyzed by a one-way analysis of variance (ANOVA) and *t*-test using GraphPad Prism 6 (GraphPad Software Inc., United States). Microarray raw data were extracted automatically in Affymetrix data extraction protocol using the software provided by Affymetrix GeneChip^®^ Command Console^®^ Software (AGCC). After importing CEL files, the data were summarized and normalized with robust multi-average (RMA) method implemented in Affymetrix^®^ Expression Console^TM^ Software (EC). We exported the result with gene level RMA analysis and performed the differentially expressed gene (DEG) analysis. The comparative analysis between test sample and control sample was carried out fold change. For a DEG set, hierarchical cluster analysis was performed using complete linkage and Euclidean distance as a measure of similarity. Gene-Enrichment and Functional Annotation analysis for a significant probe list was performed using DAVID^1^. The raw data have been registered in the ArrayExpress (EMBL-EBI) under accession number E-MTAB-5757.

## Results

### Growth under Various Stress Conditions

**FIGURE 1:**
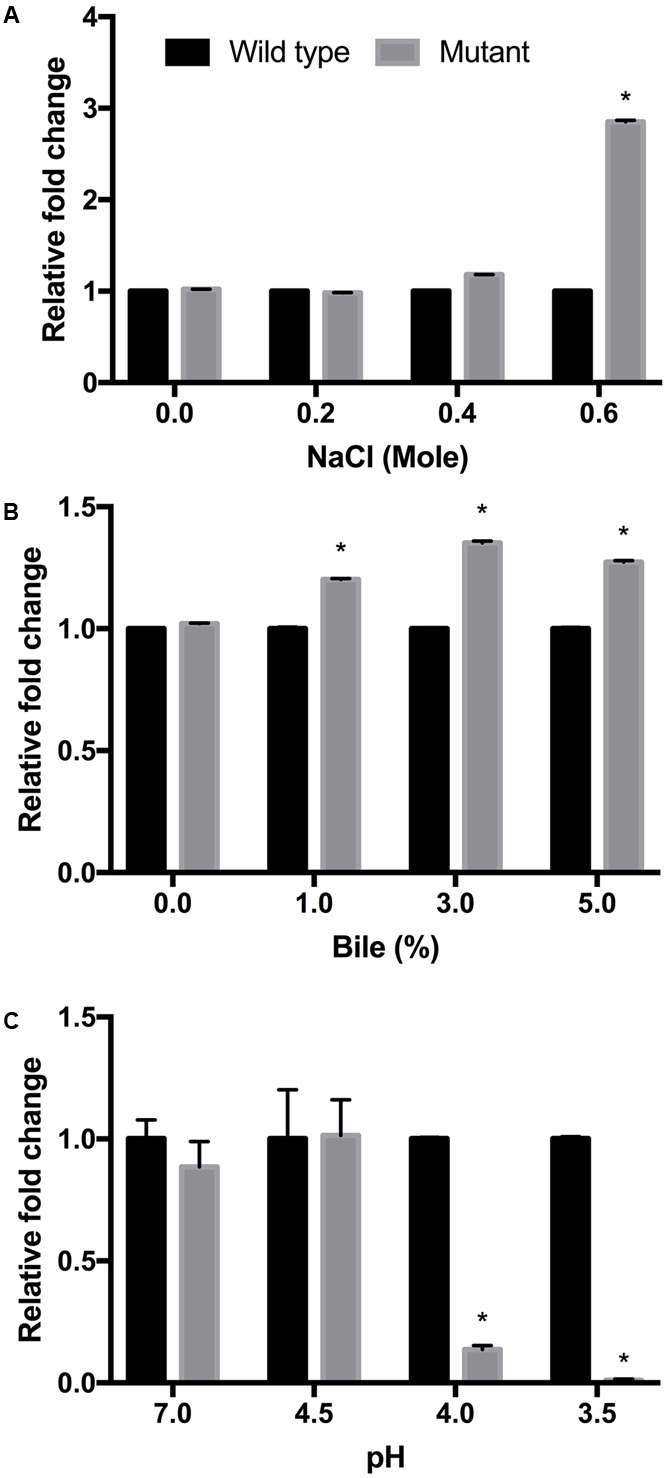
Growth of EHEC wild-type strain (ATCC 43894) and the *luxS*-deficient mutant strain under different culture conditions. NaCl **(A)**, bile **(B)**, and pH **(C)** tests were performed using LB broth at 37°C for 24 h. The fold changes were calculated from optical density values measured at 600 nm, and compared to those for the wild-type strain. Error bars indicate the standard deviations. ^∗^*p* ≤ 0.05.

Enterohemorrhagic *Escherichia coli* serotype O157:H7 wild-type and *luxS* mutant strains were cultured under various stress conditions, with clear growth differences under osmotic, bile, and acidic stress, respectively (**Figure 1**). In the case of acidic stress, the *luxS* mutant strain showed weak growth capacity with decreasing pH (**Figure 1C**). These results were consistent with those previously reported (Moslehi-Jenabian et al., 2009). In the presence of 0.6 M NaCl and >1.0% bile concentration, however, the *luxS* mutant strain showed a higher growth rate compared to the wild-type strain (**Figures 1A,B**). Osmotic stress conditions, in particular, induced significant differences in growth performance. van Kessel et al. (2015) reported *N*-Acyl homoserine lactone (AHL) quorum signaling regulates the response to the osmotic stress in *Vibrio harveyi*. They used an AHL signal regulator gene *luxR* in genetic engineered strain models in which the osmotic tolerance system glycine betaine operon *bet*IBA-*pro*XWV was induced by the quorum signaling. In this case, therefore, the *luxR* deficient *V. harveyi* strain was only weakly resistant compared to the wild-type. By contrast, in our study, the AI-2 synthase gene *luxS* deficient *E. coli* strain showed strong tolerance to the osmotic conditions applied. Temperature (25, 37, and 50°C), alkaline (pH 8.5), and anaerobic conditions were also tested as single stress factors, respectively, but no significant differences were found between the strains (data not shown).

### Responses under Osmotic Stress

**FIGURE 2:**
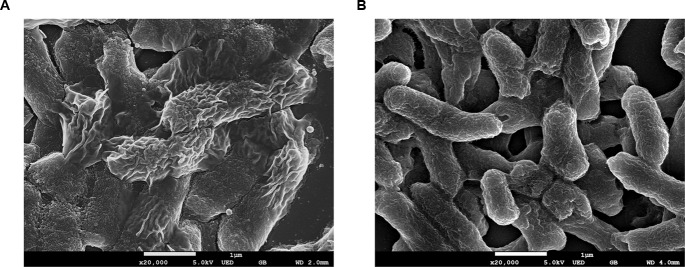
Field Emission Scanning electron analysis (FE-SEM) analysis. EHEC wild-type **(A)** and *luxS*-deficient mutant **(B)** were cultured in 0.6M NaCl LB broth for 12 h.

**FIGURE 3:**
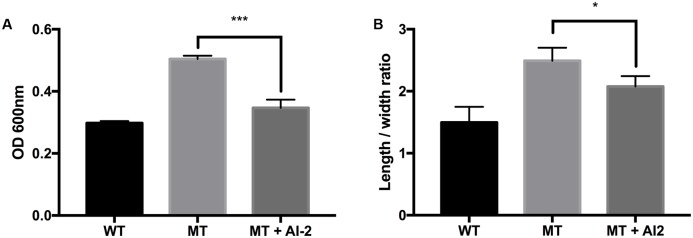
Influence of AI-2 on growth of *E. coli* wild-type strain (ATCC 43894) and the *luxS*-deficient mutant strain under osmotic stress. EHEC wild-type (WT), *luxS*-deficient mutant (MT), and the mutant with 3 μM (as final concentration) of AI-2 (MT + AI-2) were cultured for 10 h under 0.6M NaCl LB broth. The growth rates were measured from optical density 600 nm values **(A)**. Individual cell length-to-width ratio **(B)** was measured and calculated from at least 90 representative objects. Error bars indicate the standard deviations. Significance is indicated (^∗^*p* ≤ 0.05; ^∗∗^*p* ≤ 0.01; ^∗∗∗^*p* ≤ 0.001).

Morphological observation of the strains by using FE-SEM showed no differences in appearance under normal conditions. However, clear morphological differences were observed under osmotic stress (**Figures 2**, **3**). The EHEC wild-type strain showed abnormal shriveling formations, and boundaries of the outer cell membrane appeared unclear and uneven (**Figure 2A**). However, the *luxS* mutant strain did not show outer membrane surface damage, but instead, structural degradation was observed (**Figure 2B**), also when considering that bacterial cell length-to-width ratio defines its shape (Cooper, 2012). The length/width ratio also differed between the strains (**Figure 3B**). Moreover, when AI-2 was added to the mutant strain, both growth rate and length-to-width ratio were reduced (**Figure 3**). These results strongly suggest a direct influence of AI-2 on EHEC tolerance under osmotic stress, and *luxS* deficiency affecting a hitherto unknown biological reaction by which growth and/or viability are improved under specific stress conditions.

**FIGURE 4:**
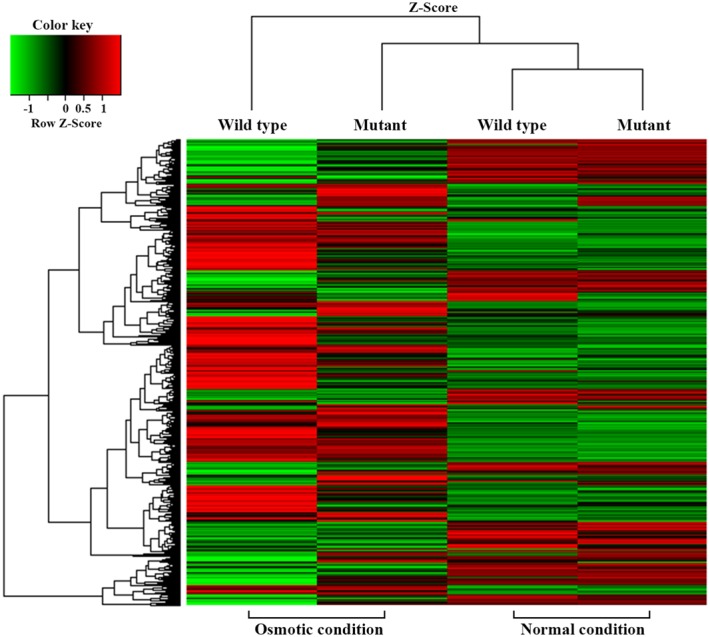
Cluster analysis using a hierarchical clustering heatmap.

For transcriptional analysis, the microarray was performed with the strains under normal and elevated osmotic conditions. Gene expression cluster comparison is indicated by the hierarchical clustering heatmap in **Figure 4**, showing differences in gene expression under different conditions. Under osmotic stress, 2174 probes of the wild-type strain were regulated (fold change > 2) compared to the normal condition, but only 1304 probes of the mutant strain. Under normal conditions, the strains showed relatively similar gene expression. Expression change in osmotic stress response genes is summarized in **Table 1**. The potassium uptake systems were down-regulated under osmotic stress in the wild-type strain. Moreover, the genes encoding the trehalose operon (*ots*AB) and glycine-betaine transporting system (*bet*IBA-*pro*XWV) that are related to tolerance to the extreme osmotic stress, were up-regulated with master regulator *rpoS* and capsule biosynthesis regulator *rcsA* in the wild-type strain. In the mutant strain, these genes were also up-regulated, but the fold changes were slightly less than in the wild-type. Furthermore, unlike the wild-type strain, the potassium transporting (KdpD) system of the mutant strain was partially activated. Other significant changes in expression of functional genes are described in **Table 2**. The strains showed differences in gene expression regulation of flagella and partial acid-resistance gene groups, but a similar regulation of chemotaxis in the osmotic stress compared to the normal conditions. In the strain comparison, however, the mutant strains showed a higher expression rate of chemotaxis, flagella, and partial acid-resistance gene groups than the wild-type strain in both osmotic and normal conditions. With regard to pathogenicity, curli and Shiga-toxin group were down-regulated in both strains by osmotic stress. While the strains showed up-regulation in hemolysis, T3SS, and biofilm formation, the mutant strain was weaker compared to the wild-type strain in agreement with previous reports (Kendal et al., 2007; Bansal et al., 2008).

**Table 1:** Gene expression changes in *Escherichia coli* O157:H7 strain ATCC 43894 in response to osmotic stress at 0.6 M NaCl.

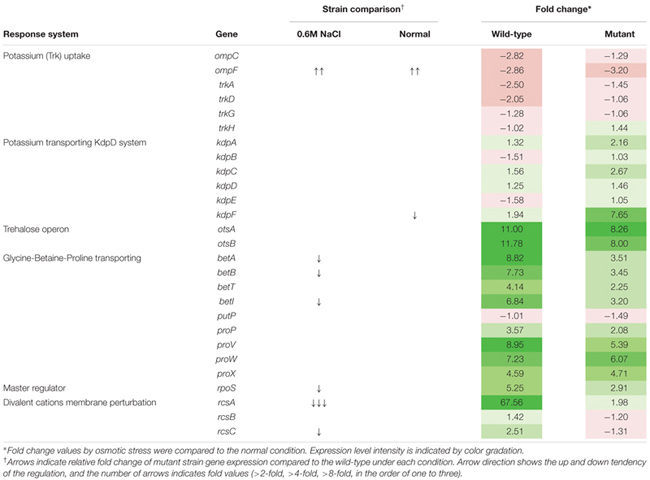

**Table 2:** Changes in environmental response and virulence gene expression of *Escherichia coli* O157:H7 strain ATCC 43894 in response to osmotic stress at 0.6 M NaCl.

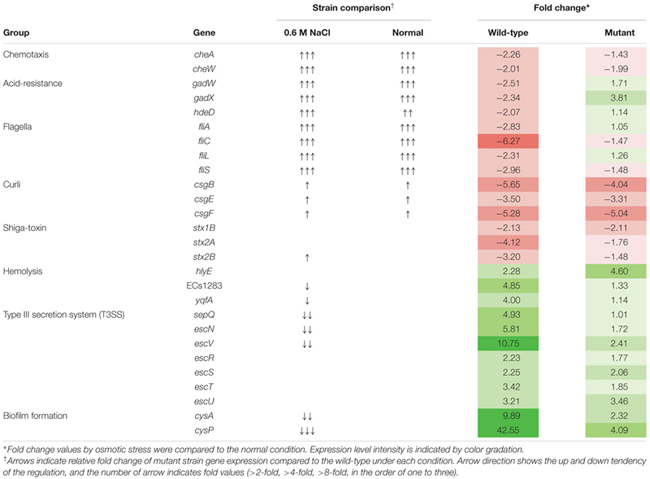

### Responses in the Gastrointestinal Environment

**FIGURE 5:**
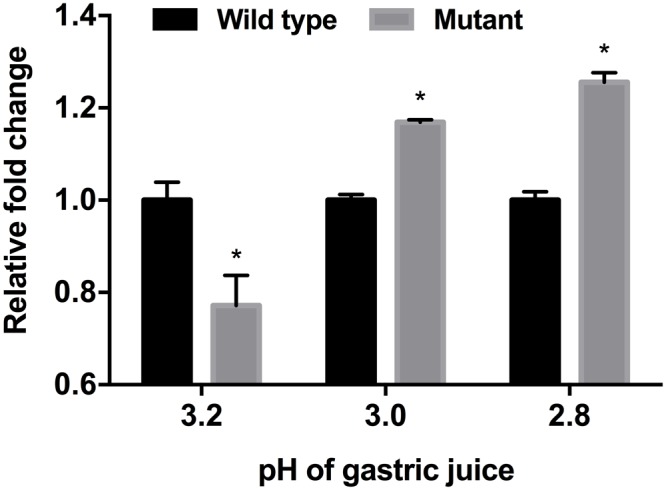
Influence of different pH-values of gastric juice on viability of EHEC wild-type strain (ATCC 43894) and the *luxS*-deficient mutant strain in an *in vitro* GIT model. ATP activity was determined and used as basis for comparison of viability of the EHEC wild-type and the *luxS* deficient mutant. The fold changes were calculated on the basis of the value of the wild-type strain. Error bars indicate the standard deviations. Significance is indicated (^∗^*p* ≤ 0.05).

**FIGURE 6:**
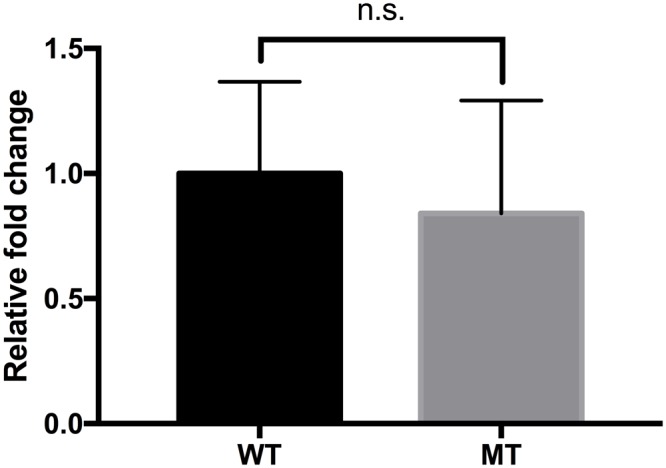
Comparison of the survival of the EHEC wild-type and the *luxS*-deficient mutant strains in the mouse gastrointestinal tract. The values were determined by FITC detection. Calculation of the fold changes was based on the value of the wild-type strain. Error bars indicate the standard deviations.

*In vitro* and mouse *in vivo* GIT murine models were performed as an extension and intensifying complex stress conditions. In the simulated *in vitro* GIT model, the results were different with regard to the pH of gastric juice (**Figure 5**). At pH > 3.2, there was no difference between the wild-type and mutant strains (data not shown). However, the *luxS* mutant strain showed a lower survival rate at pH 3.2, but a higher survival rate at pH < 3.0 compared to the wild-type strain. To examine the *in vivo* survival ability in the mouse, FITC-conjugated *E. coli* O157 antibodies were used as the reporter; however, no significant differences between wild-type and mutant infected groups could be detected (**Figure 6**).

## Discussion

### Quorum Sensing in EHEC under Stress Conditions: Signaling Status and Environmental Variables Render an Unpredictable Response

Differences in impact of stress conditions are indicated by rapid responses of bacteria to both heat and osmotic shock (in minutes) as compared to cold shock (hours) (Spano and Massa, 2006; Sugimoto et al., 2008). Quorum sensing controls certain gene expressions depending on cell density. Numerous studies have reported on quorum sensing of various pathogenic bacteria such as *Pseudomonas aeruginosa*, (Smith and Iglewski, 2003; O’Loughlin et al., 2013), *Listeria monocytogenes* (Garmin et al., 2009; Riedel et al., 2009), *Salmonella* Typhimurium (Surette et al., 1999; Choi et al., 2007), and pathogenic *E. coli* (Walters and Sperandio, 2006; Medellin-Peña et al., 2007). Pathogenicity factors such as biofilm formation, antimicrobial agent production, and virulence have been shown to be related to the quorum sensing systems of the bacterial strains studied (Ng and Bassler, 2009; Rutherford and Bassler, 2012). Therefore, pathogenic bacteria can integrate their behavior as a group with a specific threshold of quorum signals. Furthermore, quorum sensing interference does not seem to influence bacterial viability (Ren et al., 2001; Otto, 2004; Xu et al., 2006). For these reasons, quenching or inhibition of signaling has been considered as a promising strategy for combating pathogens (Rasmussen and Givskov, 2006; Brackman et al., 2011). However, we only have a few limited understanding of the influences of quorum signaling on bacterial physiology under diverse environmental conditions. In this study, we intended to demonstrate the role of the AI-2 quorum sensing system on EHEC growth and survival under specific stress conditions including *in vivo* mouse and *in vitro* GIT models. Our results showed different growth rates for the EHEC wild-type and the *luxS*-deficient mutant strains under various stress condition (**Figure 1**). In particular, it could be shown that LuxS/AI-2 quorum signaling was associated with EHEC osmotic stress tolerance (**Figure 3**). This correlation provides an extended understanding of a previous report on AHLs by van Kessel et al. (2015) that the AI-2 quorum sensing system is also involved in osmotic stress response systems. In the *in vitro* GIT model, unlike the response result in single pH stress conditions, the mutant strain showed superior survival with decreasing gastric juice pH-values from 3.0 and 2.8, but not at 3.2 (**Figure 5**). Although the mutant strain strongly expressed up-regulation of a few acid resistance genes (*gad*WX, *hde*D) in the microarray analysis (**Table 2**), it cannot clearly explain the inconsistent survival in the *in vitro* GIT model. While, under *in vivo* conditions in the model no significant differences in the survival of the tested strains could be detected, there may be two probable reasons explaining the result, being (1) unknown factors affecting survival, and/or (2) AI-2 signal ‘supporting’ from commensal bacteria in the mouse GIT. We assume that the quorum signal status may affect bacterial growth and/or survival under osmotic conditions of a strain with regard to the osmotic tolerance operon. This aspect may differ among strains and species depending on the specific quorum sensing system. In addition, the food matrix or host *in vivo* ecosystems present diverse environmental conditions, including unknown factors that may impact bacterial quorum signaling. In order to predict bacterial behavior, including (strain-related) bacterial growth and viability, it seems essential to define the interaction of the quorum signaling system and environmental conditions.

### Influence of AI-2 on the EHEC Physiology

Osmotic stress response of *E. coli* is related to proline, glycine, and betaine transporting systems. In our transcriptional analysis of the EHEC wild-type strain, the osmotic response systems and even another trehalose operon were up-regulated under 0.6 M NaCl osmotic stress. This condition was sufficient to suppress EHEC growth. Interestingly, although the wild-type strain showed higher up-regulation of the operons than the *luxS* deficient mutant strain, the growth rate was lower than that of the mutant. According to the DAVID database analysis, energy metabolism expression also reflected this result. In the mutant strain, the TCA cycle and oxidative phosphorylation gene clusters were significantly increased compared to the wild-type under osmotic stress (data not shown). We could not clarify the linkage and role of the *luxS* gene in the growth and survival due to differences in results, yet, there were a few clues. First, *luxS* deficiency and lack of the AI-2 molecule may affect global metabolic regulation. The gene *luxS* is involved in the methionine biosynthesis pathway, and its absence could cause changes in efficiency and construction of the pathway. Furthermore, *E. coli* has alternative AI-2-like signal molecule formation systems (Li et al., 2006; Tavender et al., 2008). Although the mutant cannot synthesize the AI-2 molecule, *E. coli* AI-2 associated complex Lsr family expression did not change significantly between the strains under both normal and osmotic conditions (data not shown). Therefore, we assume that the alternative pathways were activated in the mutant strain, and it may induce the different response of regulator LsrR. Former studies have reported on the LsrR complex network and global regulation in *E. coli* (Li et al., 2007; Byrd and Bentley, 2009). Moreover, under osmotic stress, *rcsA* was strongly up-regulated in the wild-type strain only (**Table 1**), while the Rcs phosphorelay system (specific to enteric pathogens/commensals) (Erickson and Detweiler, 2006) may affect growth and survival of *E. coli*. RcsA is a positive activator of colonic acid capsular polysaccharide synthesis (cps), and this *cps* operon is activated by osmotic stress or *rcsA* expression (Ebel and Trempy, 1999). Also, Thermo-resistance of *E. coli* is activated inconsistently by the presence of RcsA (Nagahama et al., 2006), and *rcs* genes are involved in the complex network affecting curli synthesis (Vianney et al., 2005). Moreover, rcsA is related to *sdiA*, a *E. coli* homolog of AHLs quorum sensing regulator LuxR (Ghosh et al., 2009), while the roles of the *sdiA* in *E. coli* physiology are universe and hitherto unveiled (Kanamaru et al., 2000; Li et al., 2007; van Kessel et al., 2015). The differences in *rcsA* expression may be a possible target for solving the inconsistent physiological responses between wild-type and mutant strains.

*Escherichia coli* cannot produce the AHL signal molecule, thus *in vitro* and *in vivo* experimental conditions represent different situations. Our results suggest a ‘neutralization’ of the survival differences of the strains in the *in vivo* mouse model (**Figure 6**). This may imply some AI-2 signaling interference by metabolites of commensal microbiota and/or compounds originating from host nutrient digestion. Further studies would be needed for clarification and for extending our present understanding. Under osmotic stress, virulence-related genes and some environmental response systems of the mutant strain showed typical regulation. Although the *luxS* mutant grew better than the wild-type under 0.6 M NaCl stress, practically none of the represented gene expressions showed any significant change or, with regard to the Type III secretion system and biofilm formation, were weaker than in the wild-type (**Table 2**). From these results, our study confirmed that lack of the *luxS* gene may improve specific stress resistance of EHEC. We suggest that environmental factors and quorum signal status of target pathogens should be taken into consideration for predicting and/or controlling EHEC strain behavior.

## Conclusion

Prediction and control of the growth are important issues in understanding behavior and responses of pathogenic bacteria. Since the discovery of pathogenic bacteria most valuable achievements from the numerous high-standing research groups have provided deeper insights in the pathogenicity/virulence issue. In some cases, however, (pathogenic) bacteria show unexpected responses even when environmental variables are well controlled. When studying bacterial physiology, but, the influence of environmental factors on bacterial quorum sensing and signaling systems probably needs more specific attention. This may provide a more reliable basis for predicting and the controlling bacterial growth under defined conditions. The study confirms and extends the correlation of quorum sensing and bacterial growth under stress conditions, and also has shown the EHEC AI-2 signal system to be strongly related to osmotic stress response.

## Author Contributions

HP and KL performed the experiments and wrote the manuscript. SY established anaerobic experiment conditions and standardized the *in vitro* GIT model. HS and WH were project leaders and provided funding and edited the manuscript.

## Conflict of Interest Statement

The authors declare that the research was conducted in the absence of any commercial or financial relationships that could be construed as a potential conflict of interest.

## Footnotes

1 http://david.abcc.ncifcrf.gov/home.jsp
